# Scraps

**Published:** 1897-03

**Authors:** 


### SCRAPS

PICKED UP BY THE ASSISTANT-EDITOR.

---

**Clinical Records** (18).—The minister of the parish used a galvanic battery in the case of a paralysed crofter. The following day a neighbour asked the patient's wife how her husband was getting on. "I 'm houping," she replied, "he'll sune be better. The minister's been giein' him a shock wi' the Calvinistic battery, an' it did him a lot o' guid!"

**Rhinology.**—Under the title of "A Humorous Séance" the *Scalpel* gives the account of a meeting of the Medical Society of the Twenty-ninth Arrondissement, Paris. Amongst the communications presented was "An essay by Dr. Ducornet, the eminent rhinologist, on 'The difference of arterial pression in each of the two nasal fossæ, right and left, with operatory deductions.' In his conclusions the author insists, with much reason, on the facts already partially recognized—that rhinology is a field far too vast for the activity of a single specialist; that it is, therefore, necessary, to divide its specialization; and that in future the illuminants of this branch of medical science should be known as *right* rhinologists and *left* rhinologists, the *septum nasi* alone being common ground for the two, though they should, as far as possible, endeavour to confine their interventions each to his own surface of that formation, even in cases where it may be considerably deflected to one or other side."—*The Journal of Laryngology, Rhinology, and Otology.*

Before the New Hampshire Medical Society Dr. O. B. Douglass, a nose and throat specialist of New York, said he had a profound respect for the all-round general practitioner, and that specialists being so from necessity were consequently eccentric, one-sided, instead of being well-balanced. He urged the importance of securing the patient's confidence, and, adding that specialists often get it in a degree to which they are not always entitled, he quoted the following lines:

> "I know his nose. He knows I know his nose.
> He said he knew I knew his nose.
> And, as he said he knew I knew his nose,
> Of course I know he knows I know his nose."

**Obstetrics, Home and Foreign** (3).—Labour seems to be an equally easy process among all people who live in a perfectly natural state. This may be accounted for by the active life which women lead among these people; all the work is done by them, so that the frame and the muscular system are developed, and the fœtus, by constant motion, may be said to be shaken into that position in which it best adapts itself to the maternal parts, into the long diameter, and once in such a position it is held there by the firm walls of the maternal abdomen, and the birth becomes easy. Moreover, they do not marry out of their own tribe or race, and the head of the child is adapted to the pelvis of the mother through which it is to pass.

There are certain differences and an increase of the difficulties of labour as civilisation is neared. In our centres of luxury people intermarry regardless of difference in race or frame of body, and the consequence is the frequent disproportion between the head of the child and the pelvis of the mother. In addition, the system suffers from the abuses of civilisation, its dissipations, and the follies of fashion. On account of the idle life led, and the relaxed condition of the uterus and abdominal walls, there is a greater tendency to malpositions; additional difficulties are presented by the weakened organisation, and the languid neurasthenic condition of the subjects in civilised communities. We do, however, sometimes find in our cities, more frequently in our rural districts, strong hardy women, who lead more active lives, and who pass through labour with an ease and rapidity much more like that displayed by their savage sisters.—Engelmann, *Labor among Primitive Peoples*, 1882, pp. 122-4.

I quote these statements without endorsing them, as much exception could be taken to some of them.

**Medical Philology** (XXI.).—From that undiscovered work *Mirivalensis in campo florum* the compiler of the *Promptorium* took "COOSTE, herbe. *Costus cujus radix dicitur costum.*" Pynson's 1499 edition read "coosta" for "costus." Mr. Way's note on the word is:

Of the various virtues of coste, which is the root of an Indian plant, the early writers on drugs give long details, and Parkinson has represented it at p. 1582 of his Herbal. In Mr. Diamond's curious MS. on the qualities of plants and spices, two kinds of coste are described, both brought from India: "þe oone ys heuy and rede, þe toþer is liȝt and noȝt bittere, and somedel white in colour;" and it is recommended to make an ointment of coste ground small with honey, excellent to cleanse the face of the freckles, and "a suffreyn remedie for sciatica, and to þe membris þat ben a-stonyed."

The *New English Dictionary* defines Cost as "the thick aromatic root of the composite plant *Aucklandia Costus*, now *Aplotaxis Lappa*, a native of Cashmere, imported as a spice by the Greeks and Romans. Thence transferred in the Middle Ages to another odoriferous plant," still known as "Costmary," which the Dictionary says is "an aromatic perennial plant, *Chrysanthemum (Pyrethrum, Tanacetum) Balsamita*, otherwise *Balsamita vulgaris*, N.O. *Compositæ*, a native of the orient region."

The signification of the latter half of Costmary has been variously interpreted. The *Century Dictionary* says that Palsgrave (1530) translates "Cost mary" by the French *Coste marine*, and this was probably the English view of its etymology. It was known that Rosemary is *Ros marinus*—"Sea-dew"—from some supposed resemblance of its blossoms to sea-spray, or because that was believed to favour its growth. A popular explanation associated Costmary with the Virgin Mary or St. Mary Magdalene. Lyte in his 1578 translation of Dodoens says it is called by some *Herba diuæ Mariæ*, and Florio (1598) gives "the herb Coaste or herb Marie" as an English equivalent of the Italian "costo." The New Sydenham Society's *Lexicon* perversely connects it with "costus amarus," an explanation described as "superfluous" by the *New English Dictionary*, which is inclined to favour the association with Mary as the original form because "the *Grant Herbier* of 15th c. has '*Herba Sancte Marie*, q. alio nomine dicitur *costus dulcem*,'" and also because several German names of the plant corroborate it.

The fourth edition (1748) of the translated Pomet's *History of Drugs*, a book which is in the Bristol Medical Library, describes the Arabian, the sweet, and the bitter Costus, and figures the Arabian variety. The author had not seen the others. According to Lewis's *Materia Medica*, 2nd ed., 1768, also in the Library, Costus was the plant described by Pomet, and Costmary the *Balsamita mas*. A similar description is found in many Pharmacopœias and other books of the 18th century.

Costmary was used to give a bitter flavour to beer, and thence obtained the name of "Alewort."

**Professional Competition.**—Suicide among practitioners of the healing art in Russia has reached alarming proportions—a distressing feature of the return being the comparative youth of the victims. The majority of them average between twenty-five and thirty-five years of age—all of them in the prime of life and full flush of their powers. An explanation of the phenomena is sought in the fact that the Russian medical man's lot is a peculiarly hard one. As in Italy, the profession is vastly overstocked in all the cities of the empire, and in consequence competition is exceptionally severe—so severe that a physician has been known to hold consultations from 8 a.m. to 11 p.m. in order to gain the reasonable income of 600 roubles (about £95) a year. Besides, the Russian municipalities, seconded by the lay press, have instituted gratuitous consultations in public ambulances, by which the wealthy city of Kiev, for example, withdraws from the legitimate fees of the profession as much as 27,000 roubles per annum. There the poverty of the practitioner is such that he has been known to give advice for 20 kopecks (8d.) for each consultation.—*Lancet.*

La misère des médecins en Allemagne est fort grande si l'on en juge par le fait suivant: La *Deutsche Tageszeitung* reproduit une lettre écrite par un médecin à son tailleur et à son associé pour leur demander un délai de paiement. Il termine ainsi: "Ce que j'aimerais le mieux, ce serait de prendre avec vous l'arrangement suivant; je m'engagerais, en échange des vêtements que j'ai reçus, à traiter les deux associés chacun pendant deux maladies à venir. Si l'un d'eux succombait un cours de la première maladie, le survivant aurait naturellement droit au traitement gratuit pour trois maladies. Je vous fais remarquer que par maladie je n'entends pas une indisposition passagère telle que rhume, coryza, etc., mais bien une maladie véritable, par exemple, une pneumonie, une fièvre thyphoïde, une hydropisie, un cancer, etc."—*L'Union médicale.*

The reason why there are so many starving doctors has been explained by a Parisian statistician, and with some degree of logic, too. He says physicians themselves are responsible for the state of affairs; they have taught their own skill to their patients, and to lady patronesses of hospitals and charities and societies. People no longer call in a doctor to bandage a wound, to vaccinate children, etc. The lay press is full of gratuitous medical advice on bronchitis, cramps, pimples, and headaches. Physicians *give* away thousands of consultations every year. These are some of the reasons why doctors are poor in Paris, and some of the reasons why they are poor in Chicago and elsewhere.—*Medical Century*, quoted in *Medical Age.*

The *Medical News* considers that hospital abuse is the cause of the hardships of the general profession, and if it increases much more the weary and struggling outside general practitioner can go home, shut himself up with his emaciated wife and starving children, and turn on the unlighted gas. No remedy is available. Experience has repeatedly shown that unanimous and persistent action on the part of doctors is impossible. Let a board of governors of any hospital or dispensary dismiss its attending staff because of a meek and proper assertion of rights, and however flagrant and arbitrary their action, other doctors will fall over each other in their haste to thrust their necks into the apparently welcome yoke.

**Medical "Bits of Old Bristol"** (2).—In 1278 the Bishop of Worcester, in whose diocese Bristol then was, made a visitation to the Monastery of St. Augustine, the church of which is now the Cathedral. He ordered that "in the infirmary food and drink was to be provided for the sick, and other things useful for them; and he forbade under a curse that any feign himself sick when he is not so, to live a dissolute life and fraudulently despise God's worship; and on the like penalty he forbad any secular persons being introduced to them, except the physician and the servants of the infirmary, nor should the friends that were in health meet there for the sake of drinking and surfeiting." (Barrett, *Hist. Bristol*, pp. 261–2). The term "physician of the infirmary" makes one at first think that this might be one of the forgeries by which Barrett was so deluded. But Mr. John Latimer tells me that it is probably only another instance of Barrett's mode of paraphrasing rather than translating the documents to which he had access. In this case he gives no reference to his authority. The order, which throws considerable light on the internal affairs of the monastery, had reference no doubt to its regular inhabitants; for although the hospitals of St. Bartholomew and St. Thomas in London had been in existence for some years for the care of the sick poor generally, there was nothing of the kind in Bristol at that time, and it would be only rarely that any strangers would receive attention in the monastic infirmary. As the ordinary residents in a monastery often numbered some hundreds, many of whom would be well advanced in years, the infirmary would never be without a large number of occupants. It formed an important part of the monastic institution, and "had a chapel annexed to it, wherein a service specially appointed for the sick was performed. It was furnished with suitable chambers, and was strewed with rushes as often as it was found necessary. A common appendage to it was a garden or court, for the recreation of the sick, and in some monasteries there was also a long enclosed gallery for the same purpose. By the Anglo-Saxon Institutes, when a monk was taken severely ill, he announced his disease to the abbot, or the whole of the brethren, and having received the benediction, he retired to the infirmary. To this the Norman decretals add, that, from the day he began to eat flesh there, he should walk with his hood on, and leaning on a staff. As soon as he was convalescent, after being shaved, he was to attend Divine service at the hour before chapter, and if there was a mass afterwards, not to offer at it. When the chapter commenced, he was to enter as soon as the affairs of the order began to be discussed, solicit pardon for having eaten meat, throw himself at the feet of the abbot after having received absolution, and return thanks to him and the convent, for the assistance which he had received from them during his sickness. By the same institutes, when a monk was sick beyond prospect of recovery, it was notified to the abbot and convent, by the infirmarer, and they immediately attended him, and gave him extreme unction, and afterwards administered the Eucharist to him; when his death approached, they went to witness his departure, and began the commendation of his soul. According to the Norman decretals, he was visited at first only by a deputation, who sprinkled, confessed, and absolved him, and then gave him extreme unction and the Eucharist. He was constantly attended by two monks, who read to him the Passion of our Lord and the Gospels, while he continued sensible; and when he was deprived of his understanding, they never ceased singing the Psalter. As soon as he appeared to be on the verge of dissolution, a servant laid a hair cloth over him, and sat watching until he was just departing, and then with the two monks, ran to the cloister door, and beat upon a table to give notice to the convent to come to him, which they accordingly did, and began a religious service, after which they again retired, certain of them remaining with him to sing the Psalter. The others again returned to perform the commendation of his soul. Manual exercise was commonly adopted in the infirmary, as a means for restoring health, and it appears that some of the patients were occasionally employed in sawing billets. It was usual for monks, in chronic infirmities, to spend their remaining days in the infirmary, where they formed a little society, and as they would thus behold each other's ailments, they would, doubtless, be induced to bear each other's burdens, and thus fulfil the law of Christ; descending quietly to the grave, and encouraging each other with the joyful hope of a blessed immortality." (Fox's *Monks and Monasteries*, 1845, pp. 175–7).

The infirmarer mentioned above was a special officer known as "infirmarius," whose routine duty it was to administer to the sick all their meals, and "to sprinkle holy water after compline on all their beds. Before matins he went round with a lantern to see if any who were able to rise remained in bed; and he was required to proclaim all negligences to the chapter. He had two brethren to assist him in taking care of the sick. The abbot, with the consent of the chapter, was to appoint such a person infirmarer as might be able, in case of sudden accident, to receive the confession of the sick." (*Op. cit.*, pp. 146-7.) In addition, he "was to provide them physic and all necessaries whilst living, and to wash and prepare their bodies for burial when dead." (Tanner's *Notitia Monastica*, ed. 1787, p. xix.). "If any one diseased with the angistrum, so called *ab angendo* (from choaking), and by another name, *windy*, from short breathing, wished to be bled, he was to announce it to the Infirmarer, who was to order the servant, to whom that office belonged, to do it; and find him a candle for it." (Fosbrooke's *British Monachism*, ed. 1817, p. 197.)

It seems from a statement by Fox (*op. cit.*, p. 309) that special consideration for the sick was a distinguishing characteristic of the Augustinians.

---

By some mysterious blunder I failed, in correcting the proof of p. 384 of the December number, to set right the third line of the quotation from the Babees Boke, and by an unmerited disaster the corrections were not properly made in a slip which was sent out with several copies of the *Journal*. The line should read—

"Fallynge at veryaunce with felow and fere."

